# Comprehensive Landscape of Prognostic Significance and Immune Characteristics of Myosins in Squamous Cell Carcinoma of the Head and Neck

**DOI:** 10.1155/2022/5501476

**Published:** 2022-04-18

**Authors:** Yuying Zhu, Shikang Zheng, Xingyou Zhai, Cheng Wang, Aijun Liu, Jun Ju, Xin Peng, Liwei Chen, Yongxia Zhang, Nan Ren, Yingli Xie, Junda Fan, Kai Zhao, Mingbo Liu

**Affiliations:** ^1^School of Clinical Medicine, Weifang Medical University, Weifang, China; ^2^Department of Otolaryngology Head and Neck Surgery, Hainan Hospital of Chinese PLA General Hospital, Sanya, China; ^3^School of Basic Medical Sciences, Weifang Medical University, Weifang, China; ^4^Department of Pathology, Chinese PLA General Hospital, Beijing, China; ^5^Department of Otolaryngology Head and Neck Surgery, Chinese PLA General Hospital, Beijing, China; ^6^Medical School of Chinese PLA, Beijing, China; ^7^The Second School of Clinical Medicine, Southern Medical University, Guangzhou, China

## Abstract

Myosin superfamily, a large and diverse family of molecular motors important for cell motility and migration, has been illustrated to play contradictory roles during the development of several kinds of tumors. However, the function and prognostic values of MYOs in head and neck squamous cell carcinoma (HNSCC) still remain largely unknown. In the current manuscript, the expression levels and clinical data of MYOs in HNSCC were investigated by online databases, including Oncomine, GEPIA, GEO, TCGA, HPA, UALCAN, Kaplan-Meier plotter, and CancerSEA; we found that the expression levels of MYO1B, MYO5A, and MYO10 were significantly elevated in HNSCC tissues, which were also correlated with the unfavorable overall survival (OS) of the patients. Furthermore, MYO1B/MYO5A/MYO10 interacting genes were identified, and the protein-protein interaction (PPI) networks were constructed by STRING and GeneMANIA. The enrichment analysis revealed that MYO1B/MYO5A/MYO10 associated genes mainly participated in cell metastasis and EMT processes, which were also confirmed by cell functional experiments. At last, the ssGSEA method was conducted to investigate the extent of immune cell infiltration, and we found that both the expression of MYO1B/MYO5A/MYO10 were closely correlated with the infiltration of immune cells in HNSCC. These findings implied that MYO1B, MYO5A, and MYO10 as novel prognostic factors for HNSCC and provided new strategy for HNSCC treatment.

## 1. Introduction

As the most frequent type of head and neck cancer and the seventh most common cancer worldwide, head and neck squamous cell carcinoma (HNSCC) presents extremely high morbidity and mortality and contributes to severe healthy burden [1]. HNSCC includes malignancies in the regions of the oral cavity, oropharynx, nasopharynx, hypopharynx, and larynx [2], and the occurrence of HNSCC is often thought to be correlated with HPV virus infection [3]. Regional neck lymph node metastasis is a prone feature of HNSCC; once evolved into distant metastasis, it will seriously affect patient survival and prognosis [4]. Therefore, there is an urgent need to develop prognostic biomarkers and to identify new therapeutic targets for HNSCC.

Myosins (MYOs) are actin-based motor proteins which translate energy from ATP hydrolysis into mechanical stress, whereas the aberrant expression of MYOs may result in abnormal cell migration [5]. Up to date, several MYO genes are identified in human genome (MYO1A-1H, MYO3A, MAO3B, MYO5A-C, MYO6, MYO7A, MYO7B, MYO9A, MYO9B, MYO10, MYO15A, MYO15B, MYO16, MYO18A, MYO18B MYO19...) [6]. MYOs mainly contain three subdomains: N-terminal head domain (motor domain) for ATP hydrolysis process, neck domain for calmodulin binding, and C-terminal tail domain for transport, and tail domain was considered indispensable in the signal transduction and membrane interaction [7].

Recent studies revealed that various MYOs played crucial roles in tumorigenesis and cancer development [8]. For instance, MYO1B was illustrated to contribute to cell proliferation, migration, and invasion and enhanced the activities of MMP1/MMP9 in cervical cancer [9]. Knockdown the expression of MYO10 alleviated tumor invasion and metabolic stress responses in glioblastoma [10]. MYO5B expression was reported to be upregulated in pheochromocytoma and paraganglioma tissues, and MYO5B mutation (p.L587P, p.G1611S, and p.R1641C) was demonstrated to be responsible for cancer cell proliferation and migration [11]. In HNSCC, increased expression of MYO1B was shown to aggravate cell migration and lymph node metastasis by enhancing cell motility [12], and silencing of MYO6 contributed to the inhibition of cell proliferation via regulation of cell cycle and apoptosis in oral squamous cells [13]. However, the expression and prognostic values of other MYOs in HNSCC have not been comprehensively demonstrated.

To the best of our knowledge, bioinformatics analysis has yet to be applied to explore the roles of MYOs in HNSCC. The present study assessed the correlation between MYO expression and the prognostic indicators of HNSCC by using various online analysis databases and identified the potential biomarkers for the treatment of HNSCC.

## 2. Materials and Methods

### 2.1. Transcriptional Data Extraction

Oncomine (https://www.oncomine.org) [14], GEPIA (http://gepia.cancer-pku.cn/index.html) [15], TCGA (https://cancergenome.nih.gov/) [16], and GEO (http://www.ncbi.nlm.nih.gov/geo; GSE31056 dataset) [17] public online databases were used for extraction of transcriptional information of MYOs in HNSCC. For Oncomine data, the thresholds were set as *p* < 0.05 and ∣log2 fold change (FC) | >2; the thresholds of *p* < 0.05 and ∣log2FC | >1 were used to screen the significantly expressed MYOs in other databases.

### 2.2. The Human Protein Atlas Database (HPA)

HPA database (https://www.proteinatlas.org/) [18] which provided antibody-based immunohistochemistry data was used for the detection of protein levels of MYOs in HNSCC tissues and normal tissues.

### 2.3. UALCAN

To investigate the expression of MYOs in cancer stages and nodal metastasis status of HNSCC, UALCAN database (http://ualcan.path.uab.edu/) [19] which was designed to analyze the expression level of genes in TCGA and the clinical data of patients was applied.

### 2.4. Kaplan-Meier Plotter Database and Receiver Operating Characteristic (ROC) Analysis

The prognostic significances of MYO expression in terms of overall survival (OS) information in HNSCC were demonstrated by utilizing Kaplan-Meier plotter database (http://kmplot.com/analysis/) through dividing patients to high or low expression groups of MYOs by auto selected best cut-off option. Diagnostic values of MYOs were elucidated by ROC analysis by using the pROC package based on clinicopathological parameters from TCGA, and area under the curve (AUC) > 0.8 was considered as an ideal biomarker for distinguishment.

### 2.5. STRING and GeneMANIA

STRING database (https://cn.string-db.org/) [20] and GeneMANIA database (http://genemania.org/) [21] were utilized to construct protein-protein interaction (PPI) networks of MYOs and identified interacted genes.

### 2.6. Correlation Analysis and CancerSEA Database

Correlation between the mRNA expression of MYOs was detected by using Pearson's correlation coefficient method and Corrplot package. CancerSEA (http://biocc.hrbmu.edu.cn/CancerSEA/) was applied for illustration of the functional states of cancer cells at the single-cell resolution [22].

### 2.7. Functional Enrichment Analysis

For functional enrichment analysis, we identified differentially expressed genes (DEGs) in MYO-high and MYO-low groups (∣log2FC | >1 and adjust *p* value < 0.05 as thresholds) based on TCGA data, and the top 150 positively associated DEGs were identified, then subjected to Gene Ontology (GO) enrichment included biological process (BP), molecular function (MF), and cellular component (CC) enrichment, as well as Kyoto Encyclopedia of Genes and Genomes (KEGG) enrichment with clusterProfiler package. For gene set enrichment analysis (GSEA) analysis, Hallmark gene set (h.all.v7.2.symbols.gmt) from MSigDB database (https://www.gsea-msigdb.org) was utilized with the clusterProfiler package visualized by ggplot2 package. Adjust *p* value < 0.05, FDR < 0.25, and ∣NES | >1 were considered significant enrichment.

### 2.8. qPCR, Transwell Assay, and Western Blot Analysis

FaDu cells (Human HNSCC cell line) were purchased from Procell Life Science & Technology (Wuhan, China) and cultured in DMEM medium containing 10% FBS (Procell, #164210-500). RNA interference, qPCR, Transwell assay, and western blot analysis were all performed as previously described [23]. Specific small interfering RNAs (siRNAs) were purchased from GenePharma (Shanghai, China) as follows: si-NC: 5′-UUC UCC GAA CGU GUC ACG UTT-3′ (Forward) and 5′-ACG UGA CAC GUU CGG AGA ATT-3′ (Reverse); si-MYO1B: 5′-GGA GAA AGU UUC AAC UAC ACU-3′ (Forward) and 5′-UGU AGU UGA AAC UUU CUC CUG-3′ (Reverse); si-MYO5A: 5′-GGA UUG UAG AUA AUG UCA AUC-3′ (Forward) and 5′-UUG ACA UUA UCU ACA AUC CAG-3′ (Reverse); si-MYO10: 5′-GCG GUA UAA GAG AAA UCA AAU-3′ (Forward) and 5′-UUG AUU UCU CUU AUA CCG CUG-3′ (Reverse). Primers for MYO1B are as follows: 5′-TCC TAC AGC AGG CTC ACA GTT-3′ (Forward) and 5′-GCC TCG TTG AAG ATG TGT GCT G-3′ (Reverse); MYO5A: 5′-CGG AAA GAC CTG GAG CAA ACT C-3′ (Forward) and 5′-TGC TGC ACG ATG CGG TGA TTG A-3′ (Reverse); MYO10: 5′-CAC TCT GCC GTA TTT CCA CAG C-3′ (Forward) and 5′-TTT GTG GAG CCA GCC TTG CTT G-3′ (Reverse); GAPDH (internal control): 5′-GTCTCCTCTGACTTCAACAGCG-3′ (Forward) and 5′-ACC ACC CTG TTG CTG TAG CCA A-3′ (Reverse). Antibodies for GAPDH (#ab8245; 1 : 10000) and N-cadherin (#ab245117; 1 : 1000) were purchased from Abcam, and E-cadherin (#14472; 1 : 1000) and Vimentin (#5741; 1 : 1000) were obtained from Cell Signaling Technology.

### 2.9. Single-Sample Gene Set Enrichment Analysis (ssGSEA)

The immune cell infiltration levels were measured by the ssGSEA method using GSVA package (http://www.bioconductor.org/packages/release/bioc/html/GSVA.html) as described [24]. Significance was determined by the Wilcoxon rank sum test and Spearman correlation method.

### 2.10. Statistical Analysis

For bioinformatic analysis, statistical analyses were carried out using the R package. Student's *t*-test and one-way ANOVA together with Tukey Kramer post hoc testing were used in the cellular functional experiments.

## 3. Results

### 3.1. Transcription Levels of MYOs in HNSCC

**Figure 1:**
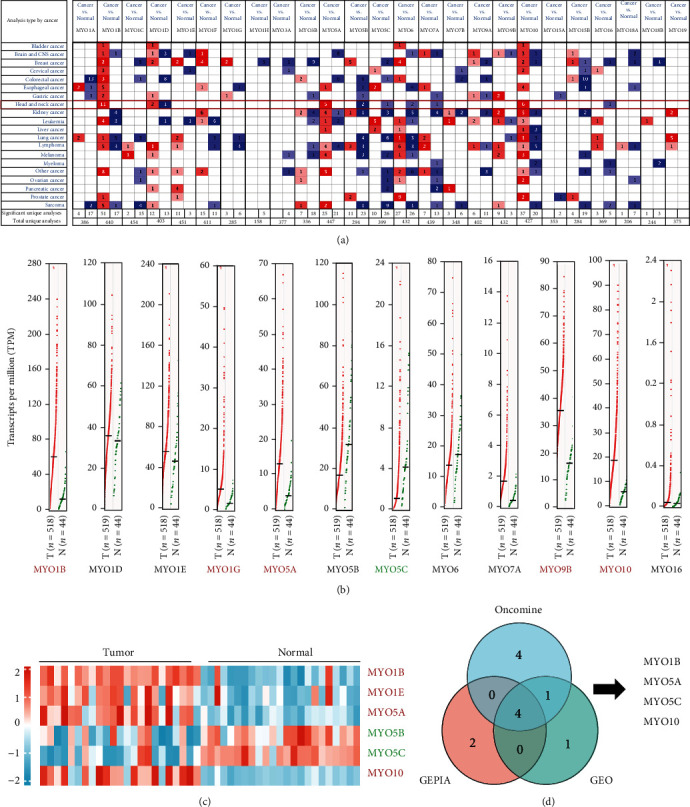
Transcription levels of MYOs in HNSCC. (a) Transcription levels of MYOs in different types of cancers in Oncomine database. Redder means higher expression, and bluer indicates lower expression. The threshold was designed with the following parameters: *p* value = 0.05, fold change = 2, and gene rank = 10%. Numbers in each cell represent the dataset numbers meeting the threshold. (b) MYO expression profiles in HNSCC patients in GEPIA database. Red means higher expression, and green indicates lower expression in HNSCC tumor tissues (*n* = 519) compared with normal tissues (*n* = 44). (c) Heatmap of expression of MYOs (thresholds: adjust *p* value < 0.05 and ∣log2FC | >1.0) in GEO database (GSE31056), the score of comparison is represented via *Z*-score. (d) Venn plot for aberrant expressed MYOs in databases (Oncomine, GEPIA, and GEO).

In order to investigate the roles of MYOs in HNSCC, Oncomine database was selected to analyze 25 MYO expressions in cancers. As shown in Figure 1(a), 11 studies were identified that reported the increased expression of MYO1B in HNSCC, 5 studies revealed the elevated level of MYO5A, and 6 studies showed the enhanced MYO10 expression in HNSCC tumor tissues compared with adjacent normal tissues. Furthermore, the levels of MYO5B, MYO5C, MYO6, MYO7A, and MYO16 were found downregulated in HNSCC patients, and 3 studies reported the aberrant expression of MYO1D: 2 showed increased expression, and 1 showed decreased expression (Figure 1(a)). In GEPIA database which contained the data from TCGA and Genotype-Tissue Expression project (GTEx), we found that the expression levels of MYO1B, MYO1G, MYO5A, MYO9B, and MYO10 were obviously increased in cancer tissues compared with normal tissues in HNSCC, whereas MYO5C was the only significantly downregulated MYO protein (Figure 1(b)). To further identify abnormal expressed MYOs in tumor and normal tissues, we compared the expression levels of MYOs in the GEO dataset. We utilized GEO2R to screen the gene expression differences between tumor and normal tissues in GSE31056 (including 23 normal samples and 23 HNSCC tumor samples). The adjust *p* value < 0.05 and ∣log2FC | >1.0 were chosen as the thresholds, and 6 MYOs were identified significantly: MYO1B, MYO1E, MYO5A, and MYO10 were found overexpressed in tumor tissues, whereas MYO5B and MYO5C expressions were alleviated (Figure 1(c)). At last, Venn analysis was performed to obtain the intersection genes among databases of Oncomine, GEPIA, and GEO, and 4 MYOs including MYO1B, MYO5A, MYO5C, and MYO10 were verified (Figure 1(d)).

### 3.2. Expression of MYO1B, MYO5A, MYO5C, and MYO10 in HNSCC

**Figure 2:**
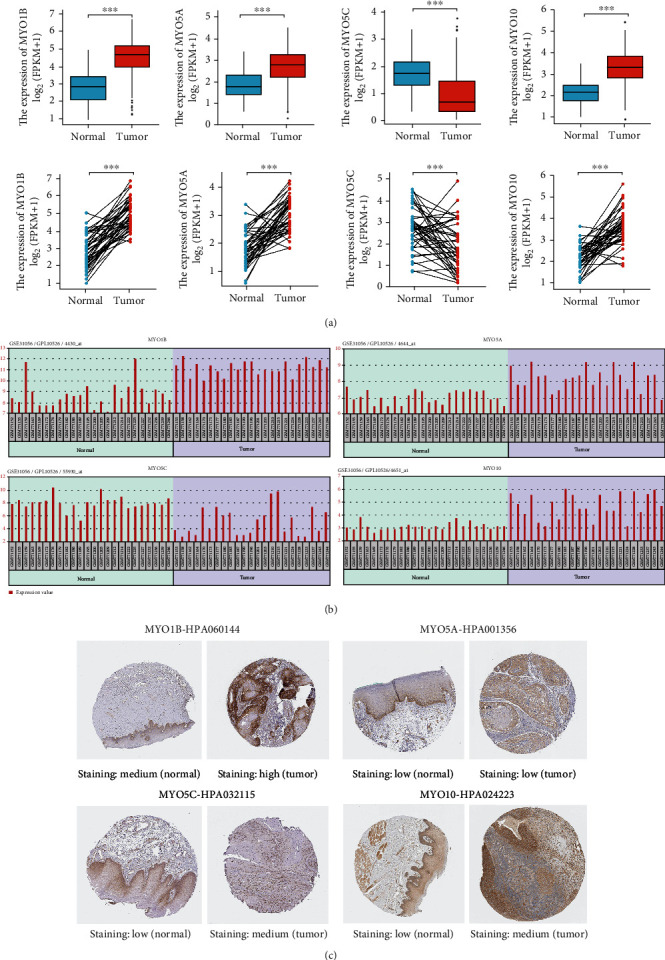
Expression of MYO1B, MYO5A, MYO5C, and MYO10 in HNSCC. (a) Comparison of expression of MYO1B, MYO5A, MYO5C, and MYO10 between tumor (*n* = 502) and normal tissues (*n* = 44) or tumor and matched normal tissues (*n* = 44 pairs) from TCGA database. (b) Expression details of MYO1B, MYO5A, MYO5C, and MYO10 in GEO database (GSE31056). (c) Protein levels of MYO1B, MYO5A, MYO5C, and MYO10 in HNSCC tissues and normal tissues (HPA database). ^∗∗∗^*p* < 0.001.

The expressions of MYO1B, MYO5A, MYO5C, and MYO10 in HNSCC tissues and matched adjacent noncancerous tissues in TCGA databases were demonstrated (Figure 2(a)), as well as the expression data in GSE31056 dataset of GEO database (Figure 2(b)). All these results confirmed the aberrant transcription levels of MYO1B, MYO5A, MYO5C, and MYO10. Furthermore, the protein levels of MYO1B, MYO5A, MYO5C, and MYO10 in HNSCC tumor tissues and normal tissues were examined by Human Protein Atlas (HPA) database; consistently, the protein levels of MYO1B and MYO10 were dramatically enhanced in tumor tissues compared with normal tissues (Figure 2(c)).

### 3.3. Prognostic Values of MYOs in HNSCC

**Figure 3:**
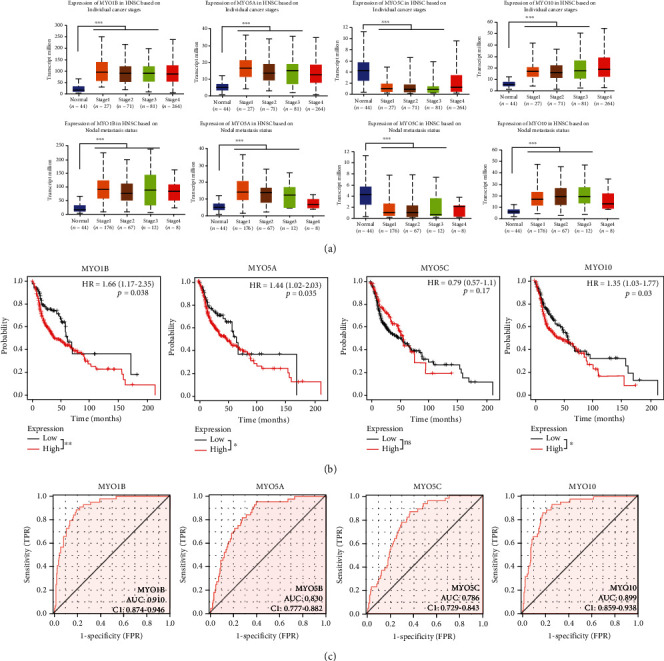
Prognostic values of MYOs in HNSCC. (a) Expression of MYO1B, MYO5A, MYO5C, and MYO10 based on individual cancer stages and nodal metastasis status in UALCAN database. (b) Correlation between overall survival data and expression of MYO1B, MYO5A, MYO5C, and MYO10 in HNSCC patients (Kaplan–Meier plotter database). (c) Diagnostic values of MYO1B, MYO5A, MYO5C, and MYO10 were shown in ROC curves based on TCGA database.

Due to the metastasis-prone nature of head and neck tumors, we examined the correlation between MYO expression and cancer stages or nodal metastasis status. As shown in Figure 3(a), MYO1B, MYO5A, and MYO10 were positively correlated with cancer stages and nodal metastasis status, whereas MYO5C which was found to be downregulated in HNSCC tissues was negatively correlated with cancer stages and nodal metastasis status. Then, we detected the prognosis values of these four MYOs, and survival analysis was performed by Kaplan-Meier plotter. We found that the increased expressions of MYO1B, MYO5A, and MYO10 were associated with poor overall survival (OS); however, the effect of MYO5C on OS was not statistically significant (Figure 3(b)). Then, we performed receiver operating characteristic (ROC) curve analysis based on TCGA data, and we found that MYO1B, MYO5A, and MYO10 had great diagnostic values for distinguishing HNSCC patients (AUC > 0.8), while the diagnostic value of MYO5C was moderately (AUC < 0.8, Figure 3(c)).

### 3.4. Correlation and Interaction Network of MYOs in HNSCC

**Figure 4:**
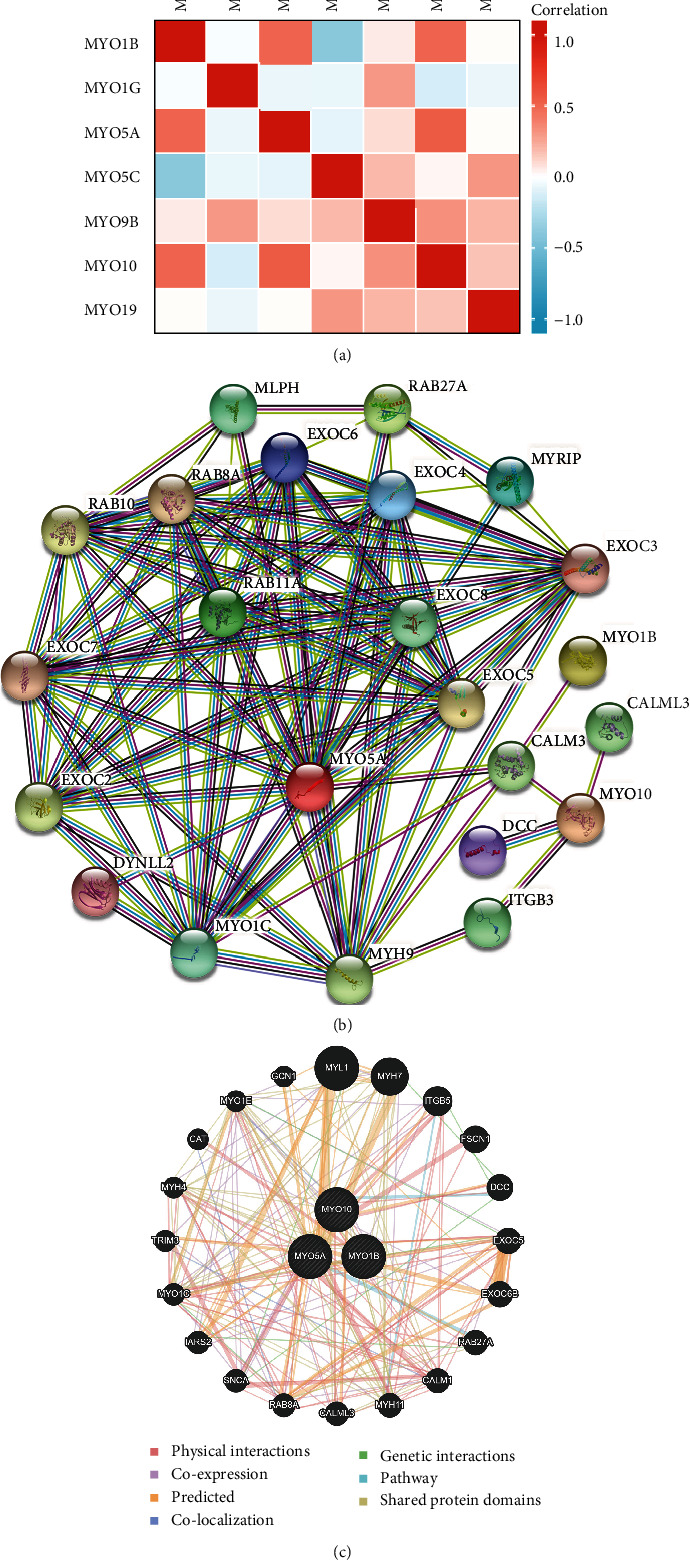
Correlation and interaction network of MYOs in HNSCC. (a) Pearson correlation analysis of individual among MYOs. (b, c) Protein-protein interaction (PPI) network for MYO1B, MYO5A, and MYO10 was constructed by STRING (b) or GeneMANIA (c).

Moreover, we examined the correlation between MYOs by the Pearson correlation method, and we found that MYO1B showed a positively correlation with MYO5A and MYO10, but negatively correlated with MYO5C (Figure 4(a)). In addition, the correlation between MYO5C and MYO5A or MYO10 was not significant. Taken together, the aforementioned details of MYO5C on expression, survival data, and diagnostic value, we considered that it may not be sufficient as a stable indicator of HNSCC; therefore, we mainly illustrate the effects and values of MYO1B, MYO5A, and MYO10 in the following studies. Due to the correlation between MYO1B, MYO5A, and MYO10, we constructed the protein-protein interaction network of the three MYOs, and 50 associated proteins in STRING database (Figure 4(b)) and 20 associated proteins in GeneMANIA database were identified (Figure 4(c)).

### 3.5. Functional States of MYOs in CancerSEA Database

**Figure 5:**
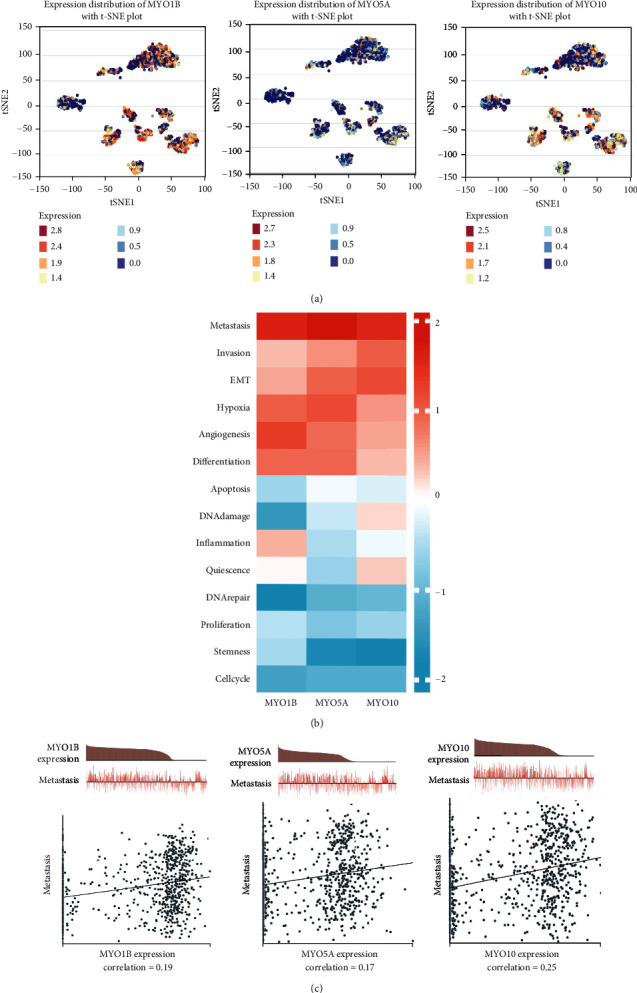
Functional states of MYOs in CancerSEA database. (a) Expression distribution of MYO1B, MYO5A, and MYO10 in HNSCC cells in CancerSEA database. Every point represents a single cell, and the colour of the point represents the expression level of MYOs. (b) Comparison of functional state analysis of MYO1B, MYO5A, and MYO10 in HNSCC cells in CancerSEA database, the score of comparison is represented via *Z*-score. (c) Correlation between expression of MYO1B, MYO5A, and MYO10 and metastasis in HNSCC cells in CancerSEA database.

After certification of MYO expression, we further explored the potential mechanisms and related biological functional processes of MYO1B, MYO5A, and MYO10. As shown in Figure 5(a), MYO expression distributions in single-cell resolution were analyzed by CancerSEA, and the cells with high expression of MYO1B/MYO5A/MYO10 were tended to cluster together, which implied the contribution of MYOs in malignant progression. Furthermore, we found that MYO1B, MYO5A, and MYO10 were positively correlated with functional states including metastasis, invasion, and EMT (Figures 5(b) and 5(c)).

### 3.6. Functional Enrichment Analysis of MYOs Associated Genes

**Figure 6:**
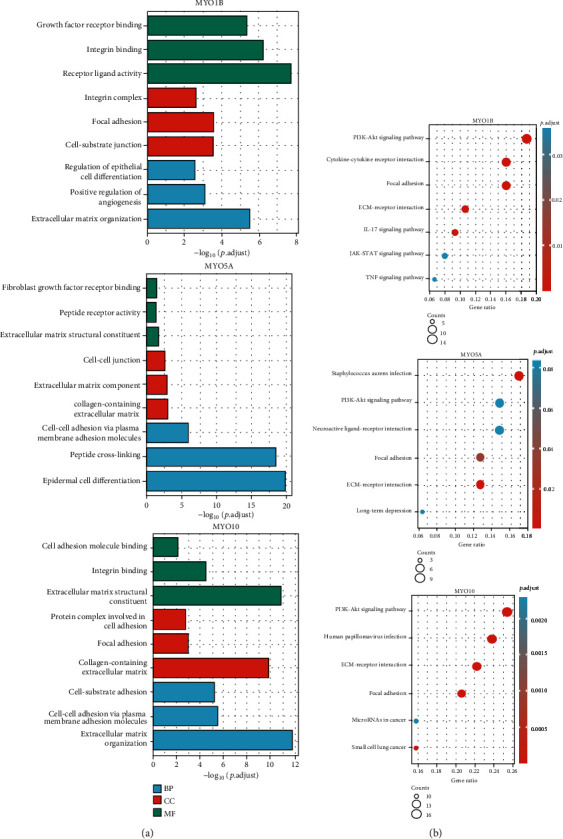
GO/KEGG enrichment of MYOs associated DEGs. (a) Gene Ontology (GO) analysis of MYO1B/MYO5A/MYO10 associated DEGs in TCGA database. (b) KEGG analysis of MYO1B/MYO5A/MYO10 associated DEGs in TCGA database. The dot indicates the gene cluster.

**Figure 7:**
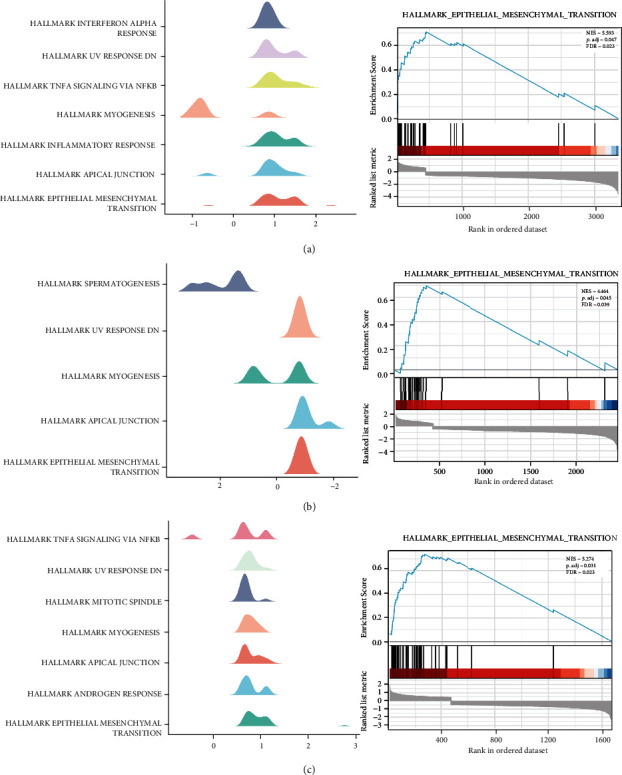
GSEA analysis of MYOs associated DEGs. (a) GSEA analysis of MYO1B associated DEGs (Hallmark gene set) and plot of “Epithelial mesenchymal transition.” (b) GSEA analysis of MYO5A associated DEGs (Hallmark gene set) and plot of “Epithelial mesenchymal transition.” (c) GSEA analysis of MYO10 associated DEGs (Hallmark gene set) and plot of “Epithelial mesenchymal transition.”

In order to evaluate the functional processes and pathways of MYOs, we identified differentially expressed genes (DEGs) in high and low MYO expression groups (criteria set of ∣log2FC | >1 and adjust *p* value < 0.05), and the top 150 positively associated DEGs were selected to perform GO (Figure 6(a)), KEGG (Figure 6(b)), and GSEA enrichment analyses (Figure 7). The involvement of MYO1B associated DEGs was mainly in the receptor and integrin binding, focal adhesion and cell-substrate junction, epithelial cell differentiation, matrix organization, etc. The enrichment of MYO5A associated DEGs was in receptor binding and peptide activity, cell junction and collagen-containing matrix, peptide cross-linking, and epidermal cell differentiation. GO enrichment of MYO10 associated DEGs was mainly involved in molecular binding and matrix constituent, focal adhesion and collagen-containing matrix, cell adhesion, and matrix organization (Figure 6(a)). In KEGG analysis results, we noticed that MYO1B, MYO5A, and MYO10 associated DEGs were enriched in PI3K-Akt signaling, focal adhesion process, and ECM-receptor interaction which were all related to tumor metastasis [25–27]. Moreover, GSEA enrichment data suggested that MYO1B (Figure 7(a)), MYO5A (Figure 7(b)), and MYO10 (Figure 7(c)) were enriched in the pathways of EMT, apical junction, and myogenesis.

### 3.7. Silencing of MYOs Inhibits Cell Migration, Invasion, and EMT

**Figure 8:**
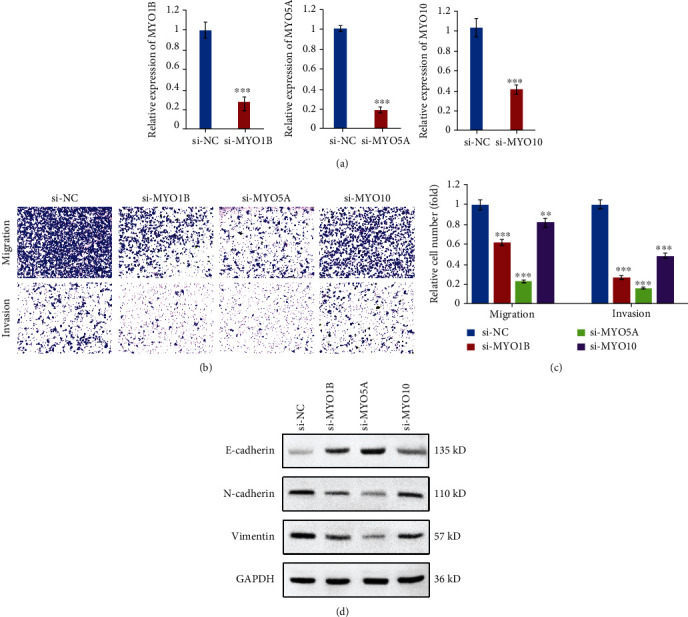
Silencing of MYOs inhibits cell migration, invasion, and EMT. (a) Efficiency of siRNAs for MYO1B/MYO5A/MYO10 was confirmed by qPCR. (b) Transwell experiments showed the abilities of migration and invasion in si-negative control or si-MYO1B/si-MYO5A/si-MYO10 treated FaDu cells. (c) Relative migrated and invaded FaDu cell number in (b). (d) Western blot analysis of the protein levels of E-cadherin, N-cadherin, and Vimentin in si-negative control or si-MYO1B/si-MYO5A/si-MYO10 treated FaDu cells. Data are presented as means ± SD of three independent experiments. ^∗∗^*p* < 0.01; ^∗∗∗^*p* < 0.001.

To verify the effect of MYOs in metastasis and EMT process, we used siRNAs specific targeted MYOs to knockdown MYO expression, and the efficiencies of siRNAs were confirmed (Figure 8(a)). Transwell assay results showed that silencing of MYO1B, MYO5A, and MYO10 significantly inhibited FaDu cell migration and invasion (Figures 8(b) and 8(c)). Furthermore, we examined the expression of EMT markers including E-cadherin, N-cadherin, and Vimentin in MYOs silenced FaDu cells, and we found that siRNA treatment greatly increased E-cadherin expression and downregulated the protein levels of N-cadherin and Vimentin, which meant that silencing of MYO1B, MYO5A, and MYO10 both alleviated the EMT potential of FaDu cells (Figure 8(d)).

### 3.8. Association between MYOs and Immune Cell Infiltration in HNSCC

**Figure 9:**
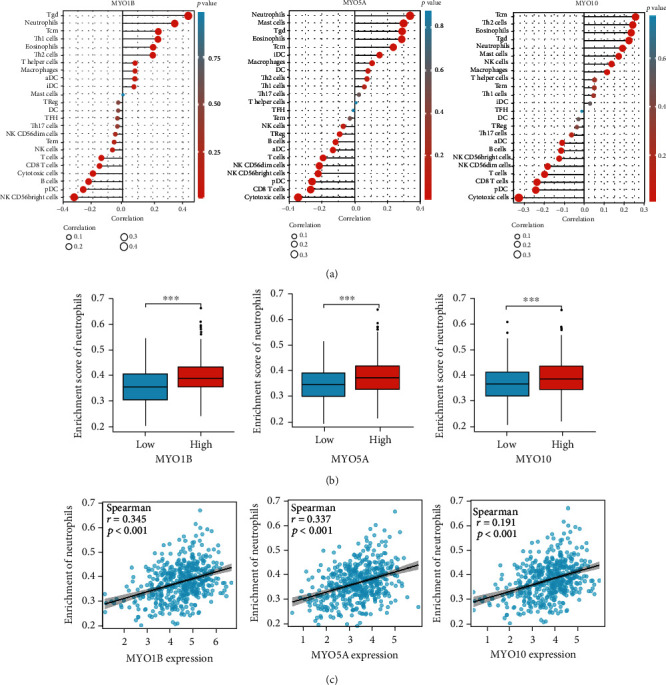
Association between MYOs and immune cell infiltration in HNSCC. (a) Correlation between the relative abundances of 24 immune cells and the expression levels of MYO1B, MYO5A, and MYO10. The size of dots denotes the absolute value of the Spearman *r*. (b) Diagrams show the difference of neutrophil infiltration enrichment between MYO1B/MYO5A/MYO10 high and low expression groups. (c) Scatter plots show the correlation between neutrophil infiltration enrichment and expression of MYO1B, MYO5A, and MYO10.

Immune infiltration and tumor immune microenvironment have been demonstrated to play essential roles in HNSCC [28, 29]. To estimate the association between MYO expression and immune cell infiltration, single sample gene set enrichment analysis (ssGSEA) was conducted. As shown in Figure 9(a), MYO1B, MYO5A, and MYO10 were positively associated with neutrophils, T gamma delta cells (Tgd), and T central memory cell (Tcm) cell infiltration, whereas negatively correlated with pDC, CD8 T cells, and cytotoxic cells. The infiltration of neutrophils was significantly aggravated in the MYO1B/MYO5A/MYO10 high expression group compared to the MYO-low expression group in TCGA database (Figure 9(b)). The correlation between MYOs and neutrophils was also shown in scatter plots (Figure 9(c)).

## 4. Discussion

Despite advances in diagnostic detection and surgical techniques, the high rates of recurrence and metastasis are still considered as mainly restricting factors in HNSCC treatment. Therefore, in-depth exploration of the crucial mechanisms and target molecules related to the development and metastasis of HNSCC is of great significance for the treatment of HNSCC. In the current study, for the first time, we comprehensively analyzed the expression details and prognostic significances of MYOs and suggested MYO1B, MYO5A, and MYO10 as effective clinical biomarkers and potential medical targets for HNSCC.

Although members of the myosin superfamily are involved in almost all aspects of human life [30], the roles of MYOs in cancers especially in HNSCC still remain to be elucidated. Benefiting from rapid advances in bioinformatics and sequencing technologies, online information databases have been significantly expanded, providing us with great help to analyze and study potential molecular markers. Previous researches revealed that MYO1B expression levels were elevated in cervical cancer [9], prostate cancer [31], colorectal cancer [32], and oral tongue cancer [12]. MYO5A was also found to be increased in glioblastoma [33] and esophageal carcinoma [34] tissues compared to the normal tissues. Aberrant levels of MYO10 were observed in breast cancer [35] as well as squamous cell carcinoma of the lung [36]. In the current study, with the utilization of databases including Oncomine, GEPIA, and GEO (GSE31056 dataset), we comprehensively analyzed 25 MYO transcription levels and obtained 4 intersection genes (MYO1B, MYO5A, MYO5C, MYO10) among these three databases. Consistent with previous reports about the elevated expression in other cancers, MYO1B, MYO5A, and MYO10 levels were increased in HNSCC tissues compared with adjacent normal tissues whereas MYO5C expression was found to be downregulated. Moreover, high expression of MYO1B, MYO5A, and MYO10 was shown to associate with the unfavorable overall survival of HNSCC patients, while the prognostic value of MYO5C was not significant as well as the moderate ROC diagnostic value. These results suggested the great potential of MYO1B, MYO5A, and MYO10 as prognostic biomarkers for HNSCC.

Recently, MYO1B was reported to aggravate colorectal cancer metastasis via enhancing rearrangement of F-actin and focal adhesion assembly mainly through targeting RhoA [32]. MYO5A was shown to be regulated by snail and contributed to cancer cell migration and invasion [37]. Cao et al. revealed that MYO10 aggravated the aggressiveness and metastasis of breast cancer cells through invadopodial formation [38]. To further explore the underlying mechanisms of MYOs in HNSCC, we used CancerSEA database to investigate MYO expression and correlated functional states. We found that the biological processes of metastasis, invasion, and EMT were extremely correlated with the levels of MYO1B, MYO5A, and MYO10. We next divided TCGA patients into high and low MYO expression groups and identified the most associated DEGs, followed by functional enrichment analysis. By synthesizing the findings of GO, KEGG, and GSEA analyses, we found similarities in the enrichment results of MYOs associated DEGs. For instance, cancer development-related PI3K-Akt signaling, focal adhesion signaling, integrin-ECM pathways, and EMT processes were all enriched in MYO1B/MYO5A/MYO10 associated DEGs. To strengthen the conclusion, we also performed cellular functional experiments, and we found that silencing the expression of MYO1B, MYO5A, and MYO10 alleviated the abilities of cell migration and invasion, and protein levels of EMT markers including E-cadherin, N-cadherin, and Vimentin were all regulated by MYO-siRNA administration.

Although the expression and functions of several MYOs in tumors have been discussed, the effects of MYOs on immune cell infiltration still remain largely unknown. Accumulating evidences suggested that immune infiltration and tumor microenvironment were extremely important in tumorigenesis, tumor development, and metastasis [39, 40]. In the current manuscript, we evaluated the association of MYO1B, MYO5A, and MYO10 expressions and immune cell infiltration, and we found that various immune cells such as neutrophils, T gamma delta cells (Tgd), T central memory cells (Tcm), pDC, and CD8 T cells were positively/negatively correlated with MYO1B, MYO5A, and MYO10 expressions, which meant that MYOs may participate in the immune processes. It is worth noting that among the immune cells, CD8 T cells and cytotoxic cells were significant negatively correlated with expression of MYO1B, MYO5A, and MYO10, suggesting that high expression of MYOs may inhibit the effect of antitumor immunity. Most importantly, we noticed that neutrophil infiltration exhibited great correlation with both expression of MYO1B/MYO5A/MYO10. Recently, neutrophils were found to release chromatin DNA filaments coated with granule proteins to form neutrophil extracellular traps (NETs) and aggravated tumor development and metastasis [41–43], which suggested that MYO1B, MYO5A, and MYO10 may associated with NET formation therefore affected EMT process and tumor metastasis.

## 5. Conclusions

In conclusion, this manuscript provided comprehensive analyses about the expression and function of MYOs in HNSCC, suggested MYO1B, MYO5A, and MYO10 as potential prognostic biomarkers in HNSCC, and revealed the potential novel roles of MYO1B, MYO5A, and MYO10 in tumor immune microenvironment.

## Data Availability

Publicly available datasets were analyzed in this study. The data used and/or analyzed during the current study are available from the corresponding author on reasonable request.
